# Comparative study of His- and Non-His-tagged CLIC proteins, reveals changes in their enzymatic activity

**DOI:** 10.1016/j.bbrep.2021.101015

**Published:** 2021-05-14

**Authors:** Daniel R. Turkewitz, Saba Moghaddasi, Amani Alghalayini, Claudia D'Amario, Hala M. Ali, Michael Wallach, Stella M. Valenzuela

**Affiliations:** a School of Life Sciences, University of Technology Sydney, Sydney, NSW, 2007, Australia; b ARC Research Hub for Integrated Device for End-user Analysis at Low-levels (IDEAL), Faculty of Science, University of Technology Sydney, NSW, 2007, Australia

**Keywords:** CLIC proteins, Oxidoreductase, Metamorphic, Moonlighting, Polyhistidine tag

## Abstract

The chloride intracellular ion channel protein (CLIC) family are a unique set of ion channels that can exist as soluble and integral membrane proteins. New evidence has emerged that demonstrates CLICs' possess oxidoreductase enzymatic activity and may function as either membrane-spanning ion channels or as globular enzymes. To further characterize the enzymatic profile of members of the CLIC family and to expand our understanding of their functions, we expressed and purified recombinant CLIC1, CLIC3, and a non-functional CLIC1-Cys24A mutant using a Histidine tag, bacterial protein expression system. We demonstrate that the presence of the six-polyhistidine tag at the amino terminus of the proteins led to a decrease in their oxidoreductase enzymatic activity compared to their non-His-tagged counterparts, when assessed using 2-hydroxyethyl disulfide as a substrate. These results strongly suggest the six-polyhistidine tag alters CLIC's structure at the N-terminus, which also contains the enzyme active site. It also raises the need for caution in use of His-tagged proteins when assessing oxidoreductase protein enzymatic function.

## Highlights

• CLIC proteins demonstrate oxidoreductase activity in the HEDS enzyme assay.
• Presence of six histidine tag on recombinant CLIC proteins alters their enzymatic activity.
• Presence of imidazole can also interfere with these enzymatic assays.
• Recommend removal of His-tags when assessing a protein's oxidoreductase activity.

## 1 Introduction

Enzymes as biological macromolecules have been extensively studied and are considered vital for the survival of all living organisms. The chloride intracellular ion channel protein family, CLICs, have been shown to have enzymatic activity [1,2] and are unusual as they exist in both monomeric soluble and integral membrane-bound states. CLIC proteins are capable of spontaneously inserting into phospholipid membranes from their aqueous soluble state where they transport both anionic and cationic species by acting as ion channel proteins; with their insertion into membranes regulated by cholesterol, amongst other factors [[3], [4], [5], [6]]. CLICs possess structural similarities to the Glutathione S-Transferase (GST) superfamily, more specifically the GST-Omega class [7,8] along with a strong homology to the Glutaredoxin (Grx) enzyme family which contain an active site dithiol motif (**Cys**-X-X-**Cys**) located in their N-terminal domain. Structural studies have shown that the soluble form of the CLIC proteins similarly display either a conserved monothiol (CLICs 1, 4, 5, and 6) or dithiol (CLICs 2 and 3) active G-site motif [1,8]. Mutagenesis studies by our group have previously shown that the monothiol active site Cysteine in CLIC1 is Cys24 [1], while CLIC3 contains a dithiol active site motif, comprised of Cys 22 [2] and Cys 25. HEDS assays were employed to demonstrate CLIC1-C24A and CLIC1-C24S had a loss of enzyme activity [1], with only a decrease in the activity for CLIC3-C22A [1,2], while the double mutant CLIC3-C22A&C25A showed almost a complete loss of enzymatic activity (Supplementary Fig. S4).

As expected from these characteristics, it has been demonstrated that CLIC1, CLIC2, CLIC3 and CLIC4 display glutaredoxin-like oxidoreductase enzymatic functions in their monomeric soluble state independent of their integral membrane ion channel activity [1,2]. As such, these recent discoveries of the enzymatic activity of CLIC family members necessitates further characterization of this activity. These moonlighting, or multi-functional, proteins likely serve as a connection or switch between multiple biochemical pathways and may function to assist in cellular responses to changes in various environmental conditions [1,9,10]. Therefore, in this study we aimed to investigate the enzymatic profile of members of the CLIC protein family using the HEDS assay system, commonly used to define several parameters such as the presence, activity, and enzyme kinetics of oxidoreductases, including CLIC, glutaredoxin, and glutaredoxin-like proteins across a variety of species [1,2,5,11,12]. The assay uses a spectrophotometric method to monitor consumption of NADPH, resulting in negative absorbances [13]. Of particular interest, we examined the effect of the six-histidine tag on the CLIC proteins’ enzymatic activity, given the common use of this tag across recombinant protein studies.

## 2 Materials and method

### 2.1 Expression and purification of recombinant CLIC1, CLIC3, and CLIC1-Cys24A

#### 2.1.1 2xYT media for bacterial growth

Standard microbial growth 2xYT medium was prepared using yeast extract (10 g), bacteriological tryptone (15 g) and NaCl (5 g) (Sigma Aldrich). The materials were dissolved in sterile water and brought to a final volume of 1 L, followed by autoclaving.

#### 2.1.2 Culture preparation, harvesting and lysing the *E. coli*

Glycerol stocks of *E.coli* BL21 (DE3) cells were transformed with the His-tagged PET28a (+) expression vector (Novagen) containing the coding sequence for either human CLIC1, CLIC3 or the mutant CLIC1-Cys24A. The bacteria were inoculated in sterile conical flasks containing 20 ml of 2xYT media with 20 μl kanamycin at a concentration of 30 mg/ml (Sigma Aldrich). The cells were left to grow overnight at 37 °C with 200 rpm shaking. Small scale bacteria cultures were upscaled into large flasks containing 350 ml 2xYT media with 30 mg/ml kanamycin and left to grow at 37 °C with 200 rpm shaking for 1.5 h. Once the bacteria achieved an optical density (OD) at 600 nm between 0.6 and 0.8, the cells were induced with 1 mM of IPTG (Isopropylthiogalactoside) at 20 °C with 200 rpm shaking for 16 h. The induced cells were harvested by centrifugation using the Hitachi high-speed centrifuge equipped with a R13A rotor at 8000 g for 30 min at 4 °C. The bacterial pellets were collected, pooled, and resuspended in 15 ml lysing buffer (300 mM NaCl, 50 mM potassium phosphate buffer pH 8.0 and 5 mM Imidazole) and then sonicated for 15–20 s cycles at 700 psi (Sonics & Materials Company/Vibra-Cell Ultrsonic Liquid Processors). Afterwards, 1 ml 20% TritonX-100 solution was added to the 25 ml bacterial homogenates. The soluble cell lysates were then collected after an additional centrifugation at 10,000 g for 40 min at 4 °C.

#### 2.1.3 Enzymatic cleavage of his-tag followed by size exclusion chromatography

The soluble fractions of the cell lysates were affinity chromatography purified using Ni^2+^-NTA beads (Qiagen). The resin slurry was loaded into an affinity column and allowed to settle, afterwards it was washed via gravity flow with 10 mL of wash buffer 1 (5 mM Imidazole, 300 mM KCl, 50 mM potassium phosphate buffer pH 8, 0.5 mM TCEP) and then 10 ml of wash buffer 2 (10 mM Imidazole, 300 mM KCl, 50 mM potassium phosphate buffer pH 8, 0.5 mM TCEP). The His-tag from the recombinant proteins were removed by thrombin enzymatic cleavage using an overnight incubation (~12 h) of bovine plasma thrombin (30 NIH units per 1L of bacterial culture) at 4 °C (Sigma Aldrich). The non-His tagged proteins were collecting in 4 × 1.5 mL fractions by washing the resin with PBS buffer (10 mM phosphate buffer, 2.7 mM KCl, 140 mM NaCl, pH 7.4, and 0.5 mM TCEP). The cleaved CLIC proteins were further purified through size exclusion chromatography (SEC) (AKTA Explorer/Amersham Pharmacia Biotech) using the HiPrep™ 16/60 Sephacryl® S-100HR column (Sigma Aldrich) with column sizing buffer (100 mM KCl, 1 mM NaN_3_, 20 mM HEPES pH 7.5 and 0.5 mM TCEP). The purified samples were stored at −80 °C. His-tagged CLIC1, CLIC1-Cys24A, and CLIC3 proteins were purified via the same SEC method with similar storage conditions. The non-His tagged CLIC1-Cys24A was generously donated [1]. Purified CLIC protein samples were quantified using the Nanodrop Spectrophotometer (ThermoFisher Scientific) using either the column sizing buffer for SEC purified proteins or elution buffer (200 mM NaCl, 250 mM Imidazole, 20 mM HEPES/pH 7.5, 0.5 mM TCEP) for affinity chromatography purified samples. Afterwards, SDS-PAGE was run using 4–15% Mini-PROTEAN® TGX Stain-Free™ Protein Gels (Bio-Rad, Australia) and visualized via Coomassie brilliant blue staining (Bio-Rad).

#### 2.1.4 Histidine tag purification and evaluation

The 6 amino acid polyhistidine tag was used in the HEDS assay as previously described [1,14] with two versions used, one in the elution buffer and the other in the column sizing buffer. Both of which had the same final concentration of 10 μM of the peptide. The elution of the histidine tag was accomplished by utilizing 1x PBS buffer to wash the CLIC proteins from the nickel column followed by further elution with 250 mM elution buffer. Afterwards some His-tag was stored in elution buffer for further evaluation. For the second condition, an ultrafiltration spin column was used to remove the entire elution buffer, and then the column was washed with column sizing buffer to remove the His-tag peptides from the membrane. The samples were stored in column sizing buffer.

#### 2.1.5 Purification of his-tagged CLIC1, CLIC3, and CLIC1-Cys24A in elution buffer

The soluble fractions were collected as previously described; however, the His-tagged fractions remained on the protein by foregoing thrombin cleavage. The His-tagged proteins were individually eluted from the nickel column by adding 2 mL elution buffer (250 mM Imidazole, 300 mM KCl, 50 mM potassium phosphate buffer pH 8, 0.5 mM TCEP). The eluted protein was stored in the elution buffer at −80 °C. In the case of His-tagged CLIC3, the same conditions were followed but different buffers were used to purify the protein: lysis buffer or wash buffer 1 (200 mM NaCl, 5 mM Imidazole, 20 mM HEPES/pH 7.5, 0.5 mM TCEP), binding buffer or wash buffer 2 (200 mM NaCl, 10 mM Imidazole, 20 mM HEPES/pH 7.5, 0.5 mM TCEP), and elution buffer (200 mM NaCl, 400 mM Imidazole, 20 mM HEPES/pH 7.5, 0.5 mM TCEP).

### 2.2 Enzyme assays

Assays were based on using a flat 96-well plate containing a final volume of 200 μl with an absorbance reading at A_340nm_ using the BioTek PowerWaveTM Microplate Spectrophotometer. All data sets were analysed using Microsoft Excel 2010 and GraphPad Prism 7. All statistics were performed using either one-way ANOVA with Tukey's multiple comparisons or the two-tailed student's *t*-test and are presented as the Mean ± S.E.

#### 2.2.1 HEDS (2-hydroxyethyl disulphide) enzyme assay

The following reagents were purchased from Sigma Aldrich: glutathione reductase (GR) from yeast, reduced glutathione (GSH), reduced nicotinamide adenine dinucleotide phosphate (NADPH), 2-hydroxyethyl disulphide (HEDS), and bovine plasma thrombin. Previous studies have demonstrated the oxidoreductase activity of the CLIC family member in the HEDS enzyme assay [1,5]. HEDS assays were performed separately for both His-tagged and non-His-tagged recombinant CLIC1, CLIC3, and the CLIC1-Cys24A proteins as described in Refs. [1,14]. The reduced monomeric His-tagged and non-His-tagged CLIC proteins were used as test proteins while HcTrx-5, obtained from parasitic nematode *Haemonchus contortus*, was used as a positive control as a known thioredoxin-like protein. HcTrx-5 was generously donated [1]. 10 μM final concentration of each protein was added to a potassium phosphate buffer (5 mM/pH 7) that contained 1 mM EDTA, 250 μM NADPH, 1 mM HEDS, and 0.5 μg/mL GR. The mixture was incubated for 5 min at 37 °C, with the reaction initiated by the addition of 1 mM GSH. The consumption of NADPH was measure at A_340nm_. A zero protein control containing either the column sizing buffer and/or the Imidazole-based elution buffer was included in each assay system.

The HEDS assay determines CLIC oxidoreductase activity via an indirect approach. The principle of the assay is that CLIC protein members have GSH-disulfide oxidoreductase or transhydrogenase activity. When the transhydrogenase activity between glutathione (GSH) and 2-hydroxyethyl disulfide (HEDS) is coupled to glutathione reductase, the reaction rate can be followed spectrophotometrically. The decrease in absorbance at 340 nm, which accompanies the oxidation of NADPH, is monitored. This reduction in NADPH is expressed as a negative absorbance and plotted as a downward slopping line. This measurement of the consumption of NADPH is an indirect measure of the CLIC protein's enzyme activity [1].

#### 2.2.2 Evaluation of his and Non-His-tagged CLIC1 and CLIC3 after SEC purification

In order to confirm whether the Histidine tag would interfere with the catalytic activity of the CLIC1 enzyme, batches of both His-tagged and non-His-tagged CLIC1 and CLIC3 were prepared as described. Both the His-tagged and non-His-tagged proteins were subjected to purification through first affinity chromatography, followed by separation using the size exclusion column, and then characterized using the standard HEDS assay (see Supplementary Fig. S1).

#### 2.2.3 Enzyme kinetics of his-tagged CLIC1 and CLIC3

Enzyme kinetics of the His-tagged CLIC1 and CLIC3 recombinant proteins were determined using varying concentrations of the HEDS substrate (0, 0.25, 0.5, 1, 2, 4, and 6 mM final concentration) with a fixed concentration of 1 mM GSH peptide and 10 μM fixed enzyme concentrations. Km and Vmax values were calculated from the Michaelis-Menten plots using Microsoft Excel 2010 and GraphPad Prism 7.

#### 2.2.4 Effect of storage buffers on the catalytic activity of his-tagged and non-his-tagged CLIC1

His-tagged and non-His-tagged CLIC1 samples were prepared as previously described where the protein sample was prepared using the ultrafiltration spin column method in order to remove the elution buffer and Imidazole content. The prepared samples were examined in the HEDS enzyme assay.

#### 2.2.5 Enzymatic activity of his-tagged and non-his-tagged CLIC1 in the presence of varying imidazole concentrations in the HEDS assay

Purified non-His-tagged CLIC1 from SEC, which was stored in column sizing buffer, and His-tagged CLIC1, stored in elution buffer, were used and evaluated in the HEDS enzyme assay. Final concentrations of 10, 20, and 50 mM Imidazole were added to the assay mixture containing 5 mM potassium phosphate buffer pH 7, 1 mM EDTA, 250 μM NADPH, 1 mM HEDS, 0.5 μg/mL GR, and 10 μM His or non-His-tagged CLIC1. His-tagged CLIC3 in column sizing buffer was used as an additional protein.

### 2.3 Molecular homology and modelling of an *in silico* his-tagged CLIC1

The human CLIC1 and CLIC3 FASTA sequence were obtained from Uniprot [15] (UniProtKB-O00299) and compared via multiple sequence alignment with the His-tagged CLIC1 and His-tagged CLIC3 sequence from the PET28a (+) expression system using Clustal Omega [16]. Due to the lack of a His-tagged protein structure in the Protein Data Bank [17], homology modelling was utilized with a combination of I-Tasser [[18], [19], [20]], trRosetta [21], and Robetta [22]. In order to ensure the highest degree of accuracy, all three prediction software algorithms were constrained using the crystallized structure of human CLIC1 (PDB ID: 1K0M/1K0N) [8]. The predicted protein structures were saved as.pdb files and imported into PyMol [23,24] for structural analysis with the wild-type CLIC1 structure and saved as PNG files.

## 3 Results

### 3.1 Analyzing the effect of column sizing and elution buffers on the enzymatic activity of his-tagged CLIC1

**Fig. 1:**
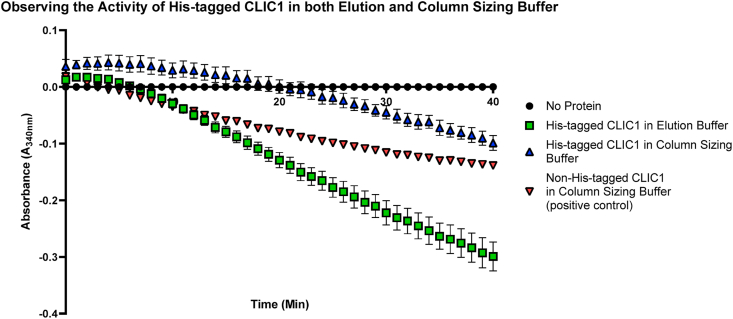
Comparison of CLIC1 protein enzymatic activity. The HEDS assay was carried out by using 10 μM His-tagged CLIC1 stored in either elution buffer or column sizing buffer along with 10 μM of the non-His-tagged CLIC1. The samples were separately added to a mixture of 5 mM potassium phosphate buffer with 1 mM EDTA, pH 7, 250 μM NADPH, 1 mM HEDS and 0.5 μg/mL GR. The mixture was heated for 5 min at 37 °C and initated via the addition of 1 mM GSH. The consumption of NADPH was monitored at A_340nm_. Results were analysed with one-way ANOVA with Tukey's multiple comparisons test. **P < 0.01, ***P < 0.001. Error bars indicate the S.E. from three independent measurements.

In the process of preparing recombinant His-tagged proteins, the elution buffer, utilized in the removal or washing of unbound proteins through the nickel affinity column, contains much higher concentrations of Imidazole (1,3-diaza-2,4-cyclopentadiene) during the final purification stage in order to release the target His-tagged protein from the affinity matrix. Therefore, it is vital that this elution buffer works efficiently to avoid alterations in the function or activity of the target protein [25]. On the other hand, gel filtration or SEC methods use the same buffer throughout the purification process. Furthermore, the buffer composition, should be compatible with the protein's subsequent storage, stability and activity [26]. As an initial comparison to analyze the effects of the two different buffers on CLIC1's enzymatic activity, purified recombinant CLIC1 proteins were run in the HEDS enzyme assay. As seen below in Fig. 1, at 40 min, the His-tagged CLIC1 and the non-His-tagged CLIC1, both stored in column sizing buffer, demonstrated similar levels of activity with an Abs reading mean of −0.099 ± 0.026 and −0.139 ± 0.003, respectively. However, the His-tagged CLIC1 stored in the Imidazole elution buffer, revealed an almost two-fold higher activity (−0.299 = ±0.57) compared to its column sizing buffer counterpart (P < 0.001) and similarly showed a significant increase in activity compared to the non-His-tagged CLIC1 control in column sizing buffer, an unexpected result (P < 0.01).

This triggered us to investigate this unusually high enzymatic activity in the imidazole-based elution buffer.

### 3.2 Purified his-tagged CLICs in elution buffer display greater oxidoreductase activity

**Fig. 2:**
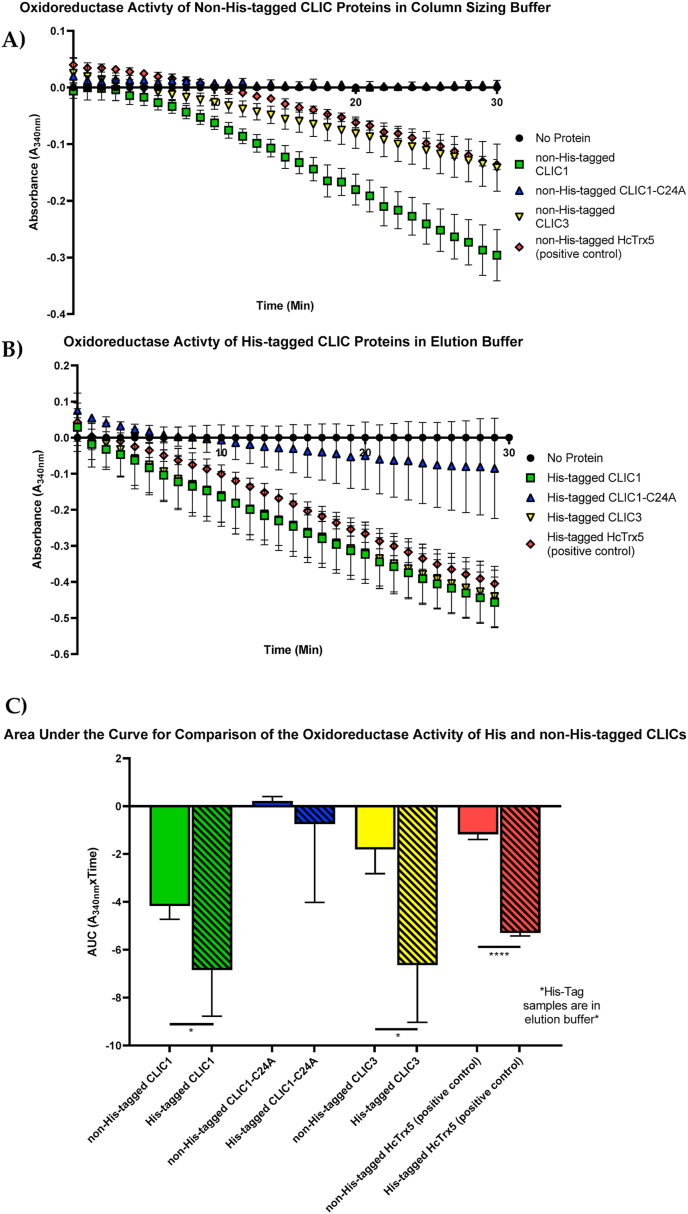
His-tagged CLIC members exhibit increased oxidoreductase activity compared to their non-His-tagged forms: **(A)** Summary of the non-His-tagged protein activity stored in column sizing buffer (100 mM KCl, 1 mM NaN_3_, 20 mM HEPES, pH 7.5 and 0.5 mM TCEP); **(B)** Summary of the His-tagged CLIC proteins stored in elution buffer (250 mM Imidazole, 300 mM KCl, 50 mM potassium phosphate buffer pH 8, 0.5 mM TCEP); **(C)** Comparison of the oxidoreductase activity of both His (in elution buffer) and non-His-tagged (column sizing buffer) CLICs. Experiments were conducted in the presence of 5 mM potassium phosphate buffer with 1 mM EDTA, pH 7 containing 10 μM His or non-His-tagged CLIC proteins or HcTrx5 (positive control), 250 μM NADPH, 1 mM HEDS and 0.5 μg/mL GR. The mixture was heated for 5 min at 37 °C and initated via the addition of 1 mM GSH. The consumption of NADPH was monitored at A_340nm_. Results were analysed with two-tailed Student's *t*-test for each protein set (His-tag vs non-His-tag). *P < 0.05, ****P < 0.0001. Error bars indicate the S.E. from three independent measurements.

To determine why the purified His-tagged CLIC proteins in elution buffer demonstrated higher levels of oxidoreductase activity, several samples from the various stages of purification were assessed for their enzymatic activity. This included samples eluted from the nickel column prior to SEC, which remained in the elution buffer containing 250 mM Imidazole. An additional set of samples was purified via SEC and buffer exchanged into the column sizing buffer containing 20 mM HEPES (pH 7.5), 100 mM KCl, 1 mM sodium azide and 0.5 mM TCEP, where all Imidazole had been removed. As seen in Fig. 2A, the purified recombinant non-His-tagged CLIC1 and CLIC3 demonstrated oxidoreductase activity in the HEDS assay, while as expected, the non-His-tagged CLIC1-Cys24A mutant did not, consistent with previously published studies [1,2]. Fig. 2B shows His-tagged CLIC1, CLIC1-Cys24A, and CLIC3 along with a non-related control His-tagged protein, HcTrx5 [27], that were all nickel column purified and stored in imidazole elution buffer. Based on the previous result and previously published data, it was expected that the His-tagged CLIC1-Cys24A mutant would lack enzymatic activity in the HEDS assay [1]. However, in this current and subsequent repeat experiments, the His-tagged CLIC1-Cys24A mutant was found to be enzymatically active, albeit much less active than the His-tagged CLIC1-wild type. Moreover, as determined by the two tailed student's *t*-test, the His-tagged CLIC1 (P < 0.05), CLIC3 (P < 0.05), and HcTrx5 (P < 0.0001) were also showing higher enzyme activity levels in the HEDS assay (Fig. 2C) compared to their non-His-tagged counterparts, which were all in Imidazole free buffer. It is evident that the His-tagged proteins in the elution buffer, when used at the same concentration, demonstrate higher enzymatic activity compared to the non-His-tagged form, in column sizing buffer.

### 3.3 His-tagged CLIC1 and CLIC3 are kinetically different to their non-his-tagged forms

**Fig. 3:**
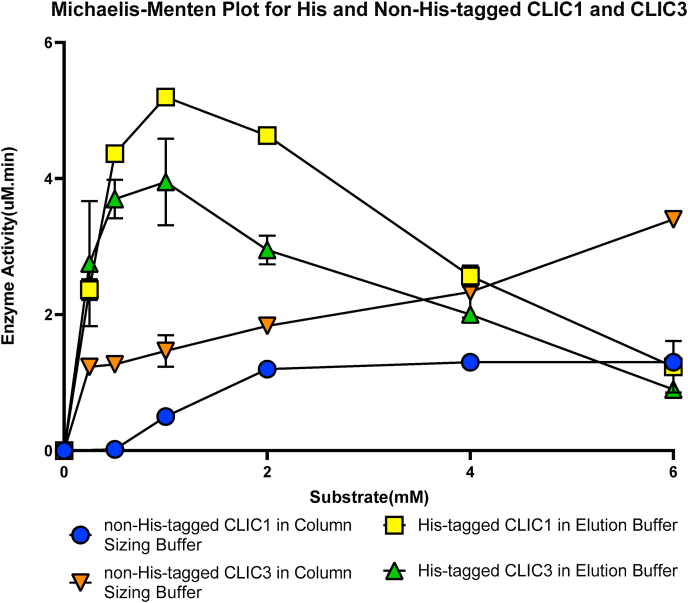
His-tagged CLIC members are not following Michaelis-Menten Kinetics. His-tagged CLIC1 and CLIC3 proteins initially display more active kinetics when stored in elution buffer with a calculated Vmax of 3.4 and 2.7 μM.min, and Km value of 0.0056 and ~1459e-016 mM, respectively. The curve reaches its peak very quickly and declines, which is not characteristic of enzymes that reach a plateau; however, the non-His-tagged CLIC1 and CLIC3 stored in column sizing buffer demonstrate Vmax of 2.026 and 3.3 μM.min and Km value of 2.503 and 0.9941 mM, respectively. This was followed by a flattening of the curve/plateau phase after reaching maximum velocity. Error bars indicate the S.E. from three independent measurements using a 10 μM final protein concentration.

Following the unexpected results from the His-tagged version of the CLIC proteins, a comparison of their enzyme kinetics were undertaken. Varying concentrations of the HEDS substrate (0.25, 0.5, 1, 2, 4, and 6 mM) were used to characterize and compare the kinetic behaviour of the His and non-His-tagged CLIC1 and CLIC3 (Fig. 3). Reminder, the His-tagged proteins were in elution buffer containing Imidazole, while the non-His tagged proteins were in sizing buffer. The results show that the His-tagged CLIC1 and CLIC3 quickly reach Vmax, followed by a slower decline inactivity. This suggests that their activity was inhibited at higher substrate concentrations, compared to their non-His-tagged forms, which show a lower maximal reaction rate by about two fold. From this result, it was evident that the His-tagged CLIC members have a greater ability to reduce the disulfide bond of the HEDS substrate once coupled with reduced GSH and Glutaredoxin Reductase (GR) in the presence of NADPH. Their activity was however inhibited at high levels of substrate and there was the confounding issue of the presence of Imidazole in the buffer. Given these results, it was decided to examine if this change was attributable to the presence of the 6-amino acid Histidine tag and then examine if other factors were at play, such as the Imidazole.

Additional assays were performed using only the non-His-tagged CLIC1 and CLIC3 proteins to demonstrate first order reactions (see Supplementary Fig. S2).

### 3.4 Activity of the histidine tag in the HEDS assay

**Fig. 4:**
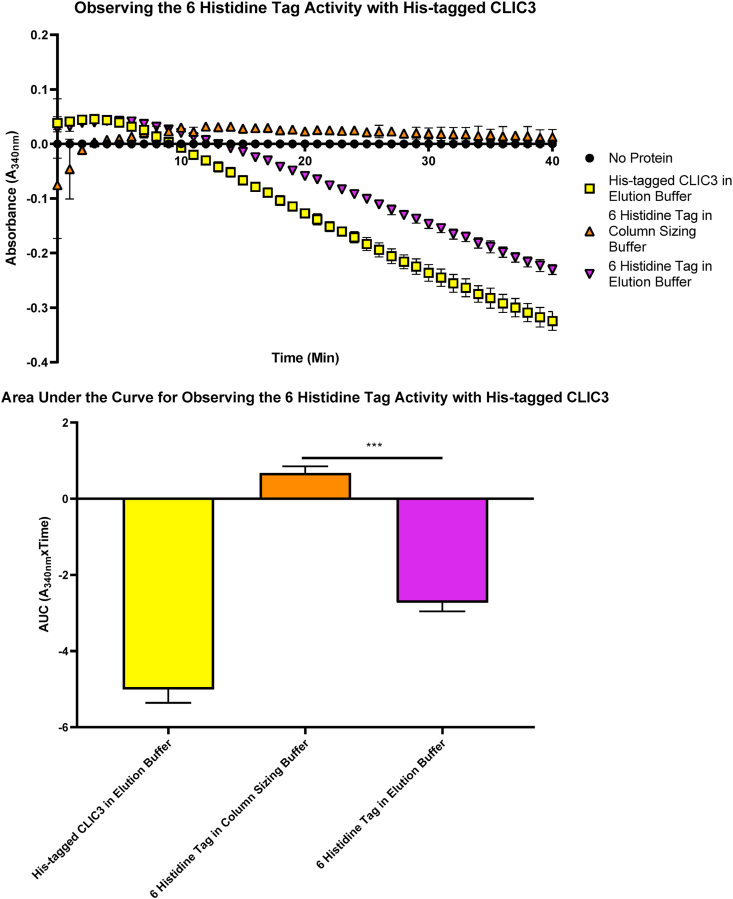
Comparison of the Poly-histidine tag peptide, in the HEDS assay. A final concentration of 10 μM of His-tagged CLIC3 (control, in elution buffer) or the 6 Histidine tag peptide in elution buffer (250 mM Imidazole, 300 mM KCl, 50 mM potassium phosphate buffer pH 8, 0.5 mM TCEP) or the 6 Histidine tag peptide in column sizing buffer (100 mM KCl, 1 mM NaN_3_, 20 mM HEPES pH 7.5 and 0.5 mM TCEP) were separately added to the mixture of 5 mM potassium phosphate buffer with 1 mM EDTA, pH 7, 250 μM NADPH, 1 mM HEDS and 0.5 μg/mL GR. The mixture was heated for 5 min at 37 °C and initated via the addition of 1 mM GSH. The consumption of NADPH was monitored at A_340nm_. Results were analysed with two tailed Student's t-test for the polyhistidine tag (in column sizing buffer vs elution buffer). ***P < 0.001. Error bars indicate the S.E. from three independent measurements.

To clarify the reason for the observed higher oxidoreductase activity of the His-tagged CLIC members, the effect of the Histidine-tag itself was investigated in the HEDS assay. In this regards, the 6 amino acid His-tag peptide was kept in either the Imidazole elution buffer (250 mM Imidazole, 300 mM KCl, 50 mM potassium phosphate buffer pH 8, 0.5 mM TCEP) or column sizing buffer (100 mM KCl, 1 mM NaN_3_, 20 mM HEPES pH 7.5 and 0.5 mM TCEP). The His-tagged CLIC3 was included as a control (stored in elution buffer) as seen in Fig. 4. From the result, it is evident that when the same 10 μM concentration of His-tagged CLIC3 or Histidine tag peptide in either elution or column sizing buffer was run in the HEDS assay, only the 6 Histidine tag peptide stored in elution buffer showed similar activity to the His-tagged CLIC3 protein, resulting in NADPH consumption at A_340nm_. In contrast, the Histidine peptide stored in column sizing buffer showed no enzymatic activity in the HEDS assay. Thus, indicating that the His-tag itself has no activity in the HEDS assay, but pointed to the Imidazole compound in the buffer as a likely contributor to the higher activity.

### 3.5 Investigation of the effect of imidazole on the enzymatic activity of his- or Non-His-tagged CLIC proteins

**Fig. 5:**
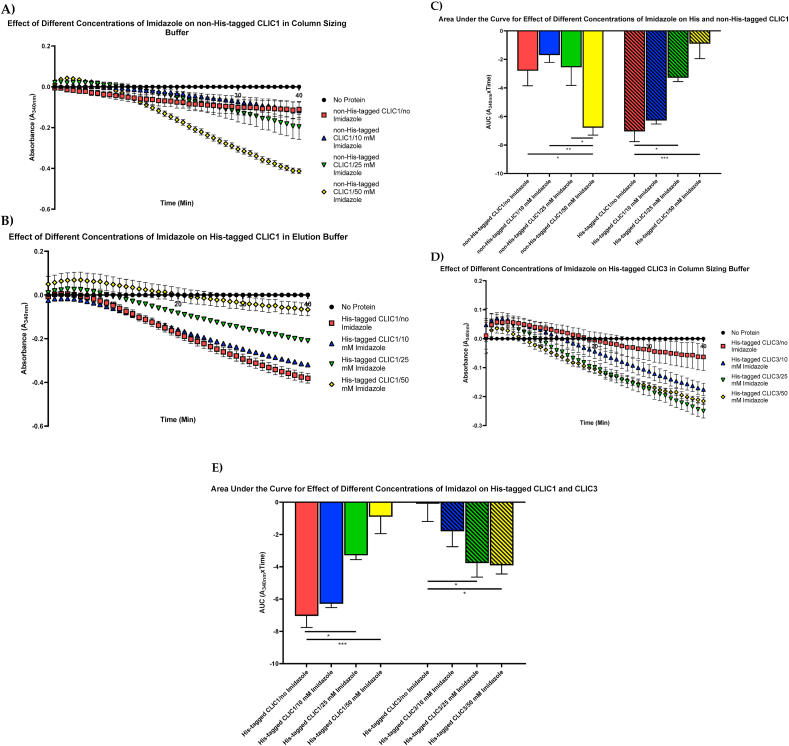
Imidazole at high concentrations interferes with the CLIC's enzymatic activity: **(A)** Non-His-tagged CLIC1, in column sizing buffer, was evaluated in the HEDS assay by the addition of increasing concentrations of Imidazole (0, 10, 25, 50 mM), that dramatically increase the enzyme's activity; **(B)** His-tagged CLIC1, in elution buffer (containing imidazole), were evaluated in the HEDS assay with the addition of additional increasing Imidazole concentrations (0, 10, 25, 50 mM) was also see to be affected by the Imidazole; **(C)** Bar graph comparison of His and non-His-tagged CLIC1. The non-His-tagged CLIC1 protein displays the highest enzymatic activity with the addition of 50 mM Imidazole while the enzymatic activity of the His-tagged isoform decreases with the increasing Imidazole concentrations; **(D)** His-tagged CLIC3, in column sizing buffer, were evaluated in the HEDS assay with the addition of increasing Imidazole concentrations (0, 10, 25, 50 mM) that raised the catalytic activity of the enzymes at 25 mM, then slightly decreased in activity at 50 mM; **(E)** Bar graph comparison of His-tagged CLIC1 (in elution buffer) and His-tagged CLIC3 stored in column sizing buffer with varying Imidazole concentrations (0, 10, 25, 50 mM). His-tagged CLIC1 significantly decreases in activity with each addition of Imidazole while CLIC3 shows an increase in activity with the addition of 10 mM Imidazole with a peak of 25 mM. Both proteins show high enzyme activity followed by a drop off in activity with CLIC1 being most significantly affected. Results were analysed with one-way ANOVA with Tukey's multiple comparisons test. *P < 0.05, **P < 0.01, ***P < 0.001. Error bars indicate the S.E. from three independent measurements.

The compound Imidazole is incorporated within many essential biological molecules such as the amino acid Histidine. Moreover, Imidazole-based Histidine complex functions in a vital role in intracellular buffering [28]. A common application of Imidazole is in the purification of His-tagged proteins in immobilized metal affinity chromatography (IMAC) systems [29]. Imidazole contained in buffers also has the ability to act as a catalyst for the cleavage of both RNA and dinucleotides [30]. From our results, it was important to investigate whether the Imidazole compound itself was interfering with the CLICs’ enzymatic activity. As seen in Fig. 5A–C, an increase in Imidazole concentration led to a significant increase in NADPH consumption in the absence of the His-tag, in contrast to the His-tagged CLIC1 proteins (in elution buffer) decreasing in activity with Imidazole. It should be pointed out, that the His-tagged proteins were in elution buffer, that already contained Imidazole, thus their base level of imidazole was higher than that of the non-His tagged proteins, resulting in higher final concentrations of Imidazole at each titration point.

It was observed that the non-His-tagged CLIC1 oxidoreductase activity is enhanced by increased Imidazole concentration with an enzymatic peak at 50 mM Imidazole. This was in contrast to the His-tagged form of CLIC1 which displayed the opposite effect and decreased in activity with the addition of Imidazole, with its activity being significantly decreased at 25 and 50 mM Imidazole (P < 0.05, P < 0.001 respectively). Fig. 5D and E demonstrated that the His-tagged CLIC1 (in elution buffer) and His-tagged CLIC3 (in column sizing buffer) proteins, were influenced by Imidazole in a different manner. The His-tagged CLIC1 significantly decreased in activity with increasing Imidazole titrations. On the other hand, CLIC3 (in column sizing buffer) showed an increase in enzyme activity with an enzymatic peak at 25 mM Imidazole (P < 0.05). Interestingly, this was also followed by a mild decrease/plateau in activity with the 50 mM titration. A prior study noted that Imidazole is known to interfere with protein interactions at concentrations of 250 mM [31]; however, the titration range used suggested changes in enzyme activity far below the 250 mM threshold.

Assays lacking HEDS substrate with varying levels of Imidazole were also undertaken to further deduce any effects of Imidazole (Supplementary Fig. S3).

### 3.6 Recombinant purified his-tagged CLIC proteins display reduced oxidoreductase activity

**Fig. 6:**
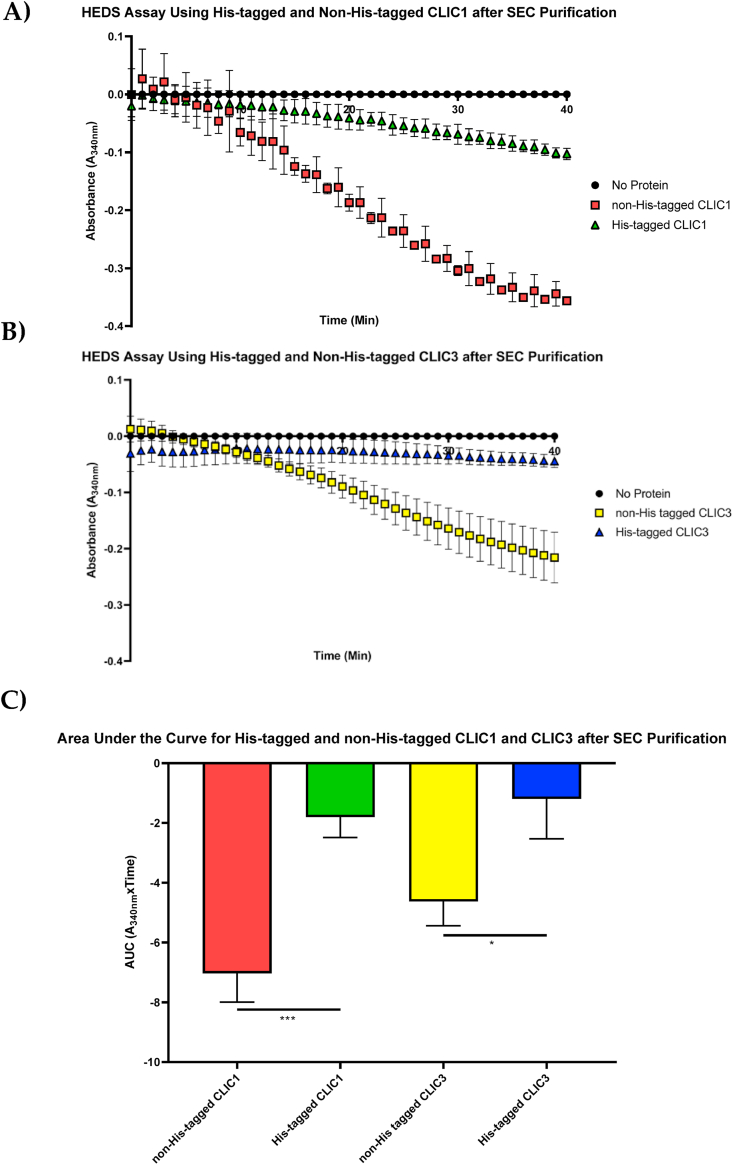
Polyhistidine-tag interferes with the catalytic activity of CLIC: **(A)** Recombinant His-tagged and non-His-tagged CLIC1 purified through SEC and stored in column sizing buffer, were evaluated in the standard HEDS assay system; **(B)** Recombinant His-tagged and non-His-tagged CLIC3 purified through SEC and stored in column sizing buffer, were evaluated in the standard HEDS assay system. The existence of the His-tag on the protein lowered its enzyme catalytic efficiency; **(C)** Bar graph comparison of His and non-His-tagged CLIC1 and CLIC3 after size exclusion chromatography and stored in column sizing buffer. The presence of the His-tag has a significant effect on the enzymatic activity of CLIC1 and CLIC3. Results were analysed with one-way ANOVA with Tukey's multiple comparisons test. *P < 0.05, ***P < 0.001. Error bars indicate the S.E. from three independent measurements.

Finally, and most importantly, to directly examine the effect of the His-tag on the proteins' enzymatic activity, we compared His-tagged and non-His-tagged CLIC proteins both stored in column sizing buffer in the HEDS enzyme assay. To achieve this, it was essential that each protein undergo the same preparation and purification procedure allowing for a direct comparison to be made. From the results obtained and shown in Fig. 6A–C, both the His-tagged CLIC1 and CLIC3 recombinant proteins, respectively, demonstrated a significant reduction in oxidoreductase activity compared to their non-His-tagged counterpart, with CLIC1 most affected by the His-tag (P < 0.001). This provides strong evidence that the six Histidine peptide tag attached to the N-terminus of either CLIC1 or CLIC3 inhibits or indirectly contributes to a reduction, in each protein's catalytic activity.

### 3.7 Molecular modelling of his-tagged CLIC1

**Fig. 7:**
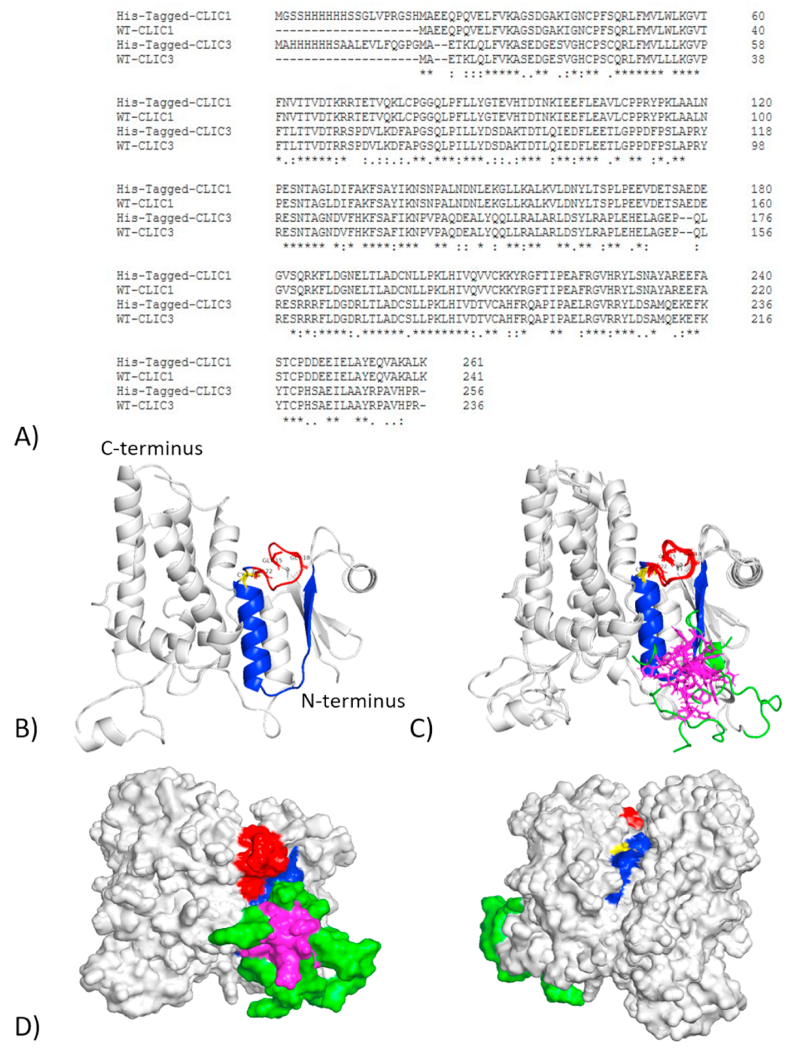
*In silico* molecular modelling of the human CLIC1 protein structure with the His-tagged CLIC1 predicted models: **(A)** Multiple sequence alignment of the wild-type CLIC1 and CLIC3 against the His-tagged CLIC1 and CLIC3; **(B)** Model of the human CLIC1 monomer (grey ribbon) with the active cysteine 24 (yellow) G^15^XXG [18]XXXG [22] cholesterol binding motif (red) and PTMD (blue); **(C)** The best predicted His-tagged CLIC1 protein structure from each prediction software was overlayed with the native CLIC1 structure. The added amino acids from the tagging process are highlighted in green with the 6-polyhistidine segment in magenta. The polyhistidine tag folds directly in front of the PTMD (blue) and directly inferior to the G^15^XXG [18]XXXG [22] cholesterol binding motif (red); **(D)** Two views related by 180-degree rotation highlighting the superimposed CLIC1 protein structures and the impact of the histidine tag (magenta) on the PTMD (blue). (For interpretation of the references to colour in this figure legend, the reader is referred to the Web version of this article.)

To elucidate what impact the addition of the His-tag to CLIC1's structure, *in silico* molecular modelling was used to predict multiple structures of a His-tagged CLIC1 [[18], [19], [20], [21], [22]]. The His-tagged models with the highest prediction scores were superimposed over the native CLIC1 structure in PyMol [8,23,24]. From the structural comparisons in Fig. 7B–D, it is apparent that the presence of the 6-polyhistidine tag is blocking the putative transmembrane domain (PTMD) as well as the key amino acids involved for cholesterol binding: Phe 31, Trp35, Phe 41, and Val43 [5]. The PTMD and hydrophobic residues are conserved in vertebrate CLIC proteins except for CLIC3 where Ile 21 is replaced by Valine, Trp35 with methionine or leucine, and Val43 with leucine [32,33]. Additionally, the presence of the additional amino acids that are introduced as part of the tagging process are also obstructing the PTMD (polyhistidine tag composing the core of the obstruction), as shown in Fig. 7D.

Interestingly, the polyhistidine tag is also located directly inferior to that of the G^15^XXG [18]XXXG [22] cholesterol binding motif previously described by our group in Hossain et al. (2019) [5]. In the same study, it was found that the mutation of either Gly 18 or Gly 22 to an Alanine resulted in a small decrease in enzymatic activity compared to the wild-type CLIC1 [5], however this was not deemed significant (P = 0.157 and P = 0.07 respectively). On the other hand, with CLIC1's active Cys24 directly adjacent to the GXXXG motif, it is not surprising that CLIC1 would lose activity similar to the CLIC1-C24A mutant used in this and other published studies that resulted in complete loss of activity [1]. However, further detailed structural studies are needed in order to confirm the precise effects of the polyhistidine tag on the protein's active site.

## 4 Discussion

The application and use of recombinant proteins across biomedical, biotechnology, biophysical and medical fields could be argued as one of the most important tools to have impacted breakthroughs in each field. Part of this success has been the widespread use of recombinant hybrids and polypeptide fusion or “affinity tags” to assist and simplify the purification and labelling process. However, these “tags” can lead to alterations in the biochemical properties of the target protein, requiring careful assessment and interpretation of results when such hybrids are used. Examples of biological processes negatively affected by the “tag” application (His, Arg, Strep tag II, FLAG tag, etc.) include, the conformational alteration in the active site of HZFB AreA protein [34], lowering the efficiency of CC49 scFv protein by negatively interfering with antigen binding [35], interfering with the post-translational modification and crystallization of SH3 domains from the chicken src tyrosine kinase [36], and the unsuccessful crystallizations of maltose-binding protein, thioredoxin and GST proteins [37]. Bucher et al. (2001) [38] set out to observe how the presence of a “tag” on crystallization was affected using the *Pyrococcus furious* maltodextrin-binding protein, which utilized five different short affinity tags (His, Arg, Strep tag II, FLAG tag and biotin acceptor peptide). From this study, it was shown that the amino acid sequence of the tag has a strong effect on both the protein's ability to form crystals and their X-ray diffraction [38]. It was further noted that the use of the Arg tag resulted in higher mosaicity and poorer diffraction compared to the non-tagged protein.

While it has been noted in a number of studies that His-tagged proteins show minimal changes in crystallization, with more than 100 structures of His-tagged proteins being deposited in the Protein Data Bank [17,38], the same effect may not be said for their biochemical properties. Throughout the purification process, native versus His-tagged proteins can differ in their mosaicity and diffraction [39,40] as a result of the presence of the 6 polyhistidine tag in spite of its relatively small size and charge [41]. A study implicated the Imidazole ring of Histidine to have effects on enzyme activity, whereas a single Histidine residue was introduced onto a Fe_3_O_4_ nanozyme surface, that resulted in an up to twenty-fold enhancement of catalytic efficiency [42]. A biochemical comparative study utilizing a His-tagged versus a non-His-tagged version of the *Staphylococcus aureus* lipase (SAL3) showed that the His-tagged SAL3 significantly changed in adsorption into CaCO_3_, compared to the wild-type, which resulted in a changed capacity to synthesize diesel additive by esterification with oleic acid [43]. It has also been observed that the presence of a His-tag affects the formation of inclusion bodies (insoluble protein aggregates), suggesting that its removal can help promote the solubility of heterologous proteins [44]. From the same study, it was suggested that the His-tag sequence may interfere with the folding of the protein in the cytoplasm when introduced into *E. coli*, providing evidence that the His-tails affect the expression, stability, or folding of recombinant proteins [44]. Furthermore, Thielges et al. (2011) [45] have shown that the attachment of a His-tag to the N terminus of myoglobin resulted in changes to the electrostatic environment of the heme pocket and dynamic of CO vibrational frequency. They concluded that the presence or absence of a His-tag should be taken into consideration when investigating protein dynamics and other properties [45]. This is contrary to many published studies and protocols that state that the His-tag, due to having a small size of ~2.5 kDa, is assumed to have no significant impact or interference on a protein's structure, function, or folding within a cell [[46], [47], [48], [49], [50], [51]].

The amino acid Histidine itself has been shown to contribute to the stabilization of proteins and conformational modifications through the alteration in entropy of its protonation in response to changes in the pH of intracellular milieu. In the case of CLIC1, it was noted that the conformational stability of the human CLIC1 protein, which structurally contains three Histidine residues, is dependent on both His 74 and His 185 that trigger the pH-induced alteration to induce a stable intermediate conformation [52,53]. The current study utilized the HEDS enzyme assay to determine alterations in the proteins’ enzymatic activity in the presence or absence of the His-tag. As shown in Fig. 6, it is very clear that the presence of the 6-polyhistidine tag on recombinant CLIC1 and CLIC3 resulted in lower glutaredoxin-like oxidoreductase activity. Additionally, the HEDS enzyme assay was performed using two different conditions, and as a result, it became clear that the His-tagged CLIC proteins stored in elution buffer demonstrated a higher oxidoreductase activity compared to their non-His-tagged counterparts (Fig. 1, Fig. 2). This effect appears to be driven by the high concentration of Imidazole in the buffer. Furthermore, as observed in Fig. 4, the 6 polyhistidine tag itself, as a discrete peptide, was separately assessed in the HEDS assay, with the peptide tested in both elution and column sizing buffer. As a result, the “tag” itself showed little to no catalytic influence when stored in the column sizing buffer, but when stored in the Imidazole elution buffer, it showed high activity compared to the His-tagged CLIC3 protein control. We therefore postulate that the high activity of the peptide tag was attributed largely to the Imidazole, which was also seen to interfere with the enzymatic activity of the CLIC proteins. This is further demonstrated by the results from Fig. 5A–E, which demonstrate the catalytic activity of both the His and non-His-tagged proteins, stored in column sizing buffer, are influenced by increasing Imidazole concentration. As the Imidazole concentration was increased, the activity of the non-His tagged CLIC proteins also increased, suggesting that the Imidazole acts as a substrate in this assay system. Future experiments incorporating the ~10 amino acid long linker between the His-tag and CLIC would be important to ascertain if the linker region also interferes with enzyme activity, distinct to the polyhistidine sequence.

Fig. 6A–C shows the activity of non-His-tagged CLIC1/CLIC3 compared directly to their His-tagged counterparts. When all proteins were handled equally and stored in the same column sizing buffer, it was revealed that the His-tagged protein had an almost two-fold lower activity. From this, we deduced that the 6 polyhistidine tag has an inhibitory effect on the oxidoreductase activity of CLIC, as previously shown with other functional properties of fusion proteins [35,54]. While it is unclear if the His-tag is occluding binding sites and or/inducing artificial conformational changes to CLIC's structure, we postulate that the “tag” interferes with the local structure surrounding the PTMD, GXXXG motif, and enzymatic active site of the CLIC protein, thus potentially impacting the structure, and lowering the enzymatic efficiency (Fig. 7). These predictions are supported by our *in silico* molecular modelling studies described above in Section 3.7. Further support of this is seen via the results in Fig. 3, where the His-tag CLIC members displayed non-Michaelis Menten like kinetics. As their curves quickly reach an enzymatic peak and then decline, rather than plateauing. However, additional experiments would need to be performed to address conformational and thermal stability changes in the presence/absence of the His-tag.

The human CLIC proteins are intriguing due to their unusual properties, which includes their dual cellular localization as both soluble globular proteins and integral membrane proteins. Due to the original discovery of bovine p64 by the Al-Awqati group [55] in their search for the chloride ion channel in cystic fibrosis pathophysiology, a large body of subsequent research on the CLICs has focused on the proteins as membrane ion channels [6,[56], [57], [58], [59], [60], [61], [62], [63], [64], [65], [66], [67], [68], [69], [70], [71], [72], [73]]; however, *in vitro* studies have now revealed that in their soluble form, they also possess oxidoreductase enzymatic activity [1,2]. It is important to note that former biophysical and electrophysiological studies of the CLIC proteins where the His-tag was present, have not reported any change in their biophysical, conformation/thermal stability or ion channel properties. Therefore, it would be pertinent for specific future studies to be undertaken to interrogate these aspects more fully. As such, it remains to be seen if the His-tag only impacts the soluble form of CLICs, as the membrane-bound form follows a different structural arrangement at its N-terminus.

## 5 Conclusion

This study focused on characterizing the CLICs' newly found oxidoreductase capabilities and specifically assessed the effect of the 6 polyhistidine tag and Imidazole compound on the oxidoreductase activity of purified recombinant CLIC protein members. His-tagged proteins displayed a decrease in their oxidoreductase enzymatic activity compared to equivalent non-His-tagged counterparts, when the proteins were run in the HEDS assay, with SEC sizing buffer. However, when the proteins were run in the HEDS assay in buffer containing imidazole (elution buffer or addition of imidazole), the His-taggged CLIC proteins showed greater enzymatic activity, compared to non-His tagged proteins. Thus, the higher enzyme activity levels were attributed to the elution buffer that contained high concentrations of Imidazole. It is therefore recommended that recombinant proteins be stored in an appropriate storage buffer lacking Imidazole to avoid interference by the imidazole in enzymatic characterizations. Importantly, the purified His-tagged CLICs following purification by SEC demonstrated reduced enzymatic activity compared to the non-His-tagged counterpart. This indeed demonstrated a direct influence on the protein's enzymatic activity due to the presence of the His-tag and supports the need for such tags to be removed from recombinant proteins prior to their use in such assay systems.

## Author contributions

Conceived and designed the experiments: S.M.V., M.W., S.M., and D.R.T. Protein expressed and purified: D.R.T, S.M, A.A, C.D. Performed the experiments: D.R.T, S.M, A.A, C.D. Analysed the data: D.R.T, S.M, A.A. Contributed reagents/materials/analysis tools: S.M.V., M.W, H.M.A., C.D. Wrote the article: D.R.T., S.M., and S.M.V.

## Declaration of competing interest

The authors declare that they have no known competing financial interests or personal relationships that could have appeared to influence the work reported in this paper.
