# Supplementary material for: Comparative study of His- and Non-His-tagged CLIC proteins, reveals changes in their enzymatic activity
Source: Biochem Biophys Rep. 2021 May 14;26:101015. doi: 10.1016/j.bbrep.2021.101015 (PMC8138732; doi:10.1016/j.bbrep.2021.101015)
Supplement: Multimedia component 1 [file mmc1.docx]

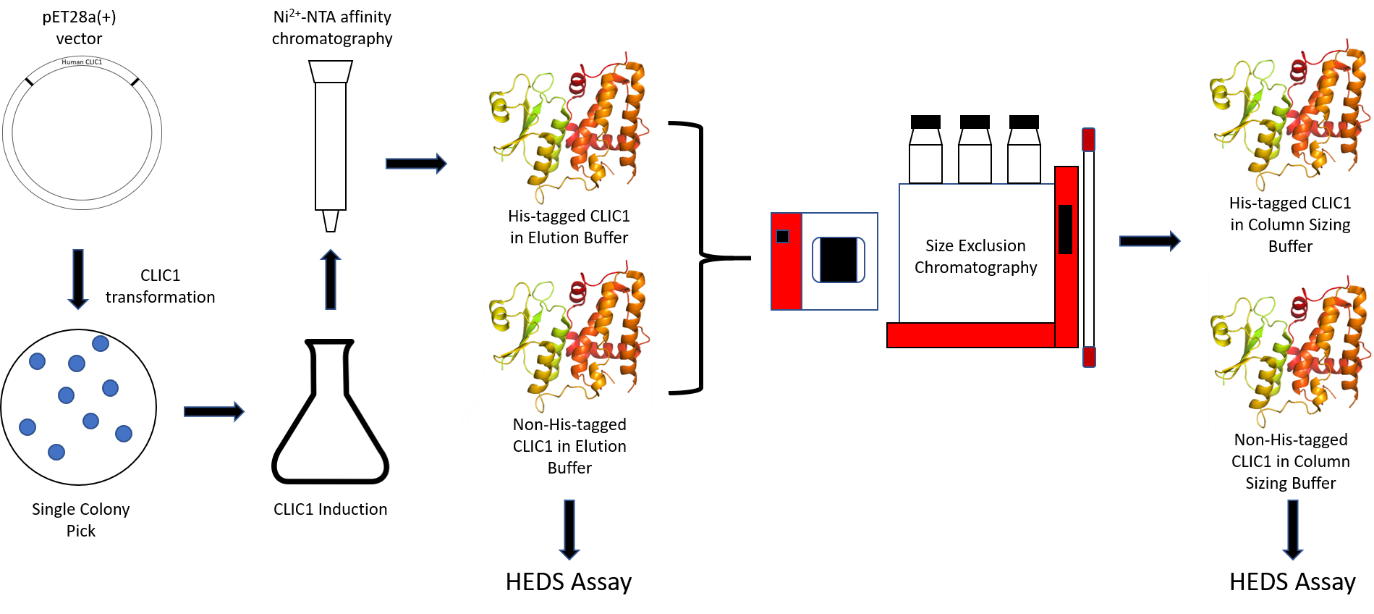


**Supplementary Figure S1.** **CLIC Protein Purification Process.** Flow chart demonstrating the overall CLIC protein purification process, highlighting the change in purification type (affinity vs size exclusion) and the storage buffers used for the HEDS enzyme assay.

**Supplementary Figure S2.** Non-His-tagged CLIC1 and CLIC3 proteins demonstrate first order reaction kinetics. A final concentration of 0, 5, 10, 15, 20, and 25 uM non-His-tagged CLIC1 or non-His-tagged CLIC3 was used. Error bars indicate the S.E. from three independent measurements.

 **Supplementary Figure S3.** HEDS free column buffer containing different concentrations of Imidazole (0 mM, 50 mM, 100 mM, 200 mM, 400 mM Imidazole) does not alter the activity of His-tagged CLIC1 or non-His-tagged CLIC1 in the HEDS assay. **(A)** Observing the activity of His-tagged CLIC1 in HEDS free column size buffer with different concentrations of Imidazole; **(B)** Observing the activity of non-His-tagged CLIC1 in HEDS free column size buffer with different concentrations of Imidazole. Experiments were conducted in the presence of 5mM potassium phosphate buffer with 1 mM EDTA, pH 7 containing 10 uM His or non-His-tagged CLIC proteins, 250 uM NADPH, 1 mM HEDS and 0.5 ug/mL GR. The mixture was heated for 5 minutes at 37°C and initiated via the addition of 1 mM GSH. The consumption of NADPH was monitored at A_340nm_. Error bars indicate the S.E. from three independent measurements.

**Time (mins)**

**Absorbance (340 nm.)**

Supplementary Figure S4: Comparison of the oxidoreductase activity of CLIC3 (WT) monomer, CLIC3-Cys22A and CLIC3-Cys22A&25A mutants and IS5(HcTrx5). A reaction mixture of 5mM potassium phosphate (pH 7) with 1mM EDTA buffer containing 250μM NADPH, 50nM GR, 1mM HEDS and 10μM CLIC3 (WT) monomer, CLIC3-C22A and CLIC3-Cys 22,25A that was incubated for 5 mins at 37ºC. The reaction was initiated with the addition of 1mM GSH and the absorbance of NADPH was monitored at A340nm. Error bars represent the standard error of at least three experimental repeats.
